# Protective Effects of Tao-Hong-Si-Wu Decoction on Memory Impairment and Hippocampal Damage in Animal Model of Vascular Dementia

**DOI:** 10.1155/2015/195835

**Published:** 2015-03-02

**Authors:** Lan Han, Zhaojie Ji, Weidong Chen, Dengke Yin, Fan Xu, Shanshan Li, Fangfang Chen, Guangyu Zhu, Daiyin Peng

**Affiliations:** ^1^School of Pharmacy, Anhui University of Traditional Chinese Medicine, Xiangshan Road, Hefei 230012, China; ^2^Department of Pharmacy, Maanshan Central Hospital, No. 27, Hudong Bei Road, Maanshan 243000, China

## Abstract

Tao-Hong-Si-Wu decoction (TSD) as a traditional chinese medicine (TCM) has been developed to treat thrombotic diseases for hundreds of years, and vascular dementia (VD) is a cognitive dysfunction syndrome caused by cerebral embolism. In this study, the protective effect of TSD on memory impairment and brain damage in rat model of VD induced by middle cerebral artery occlusion (MCAO) was investigated. The study showed that rats in MCAO treatment with TSD for 14 days significantly improved behavioral function, increased densities of neuron, and induced angiogenesis in the brain compared with model rats. TSD also adjusted the neurotransmitter levels, reduced the content of endothelin-1 (ET-1), and induced the activities of vascular endothelial growth factor (VEGF) in hippocampus. Moreover, the immunohistochemical staining and western blotting results also revealed that TSD decreased apoptosis via upregulated B-cell lymphoma-2 (Bcl-2)/Bcl-2 associated X protein (Bax) ratio. These results demonstrated TSD possesses neuroprotective and antidementia properties by preventing the loss of neural cells, adjusting brain neurotransmitter, promoting cerebral blood circulation, and decreasing apoptosis. These results suggested that TSD might be developed as an effective drug for the prevention of VD.

## 1. Introduction

VD defined a syndrome characterized by acquired mental dysfunctions resulting from brain damage of cerebrovascular origin, which is the second most common dementia in elderly, after Alzheimer's disease [1]. Epidemiological studies have shown that the prevalence of vascular dementia is in accompany with the dramatic increase in aged population, and it accounts for 20 to 30% of all dementia cases; it affects the life quality of both patients and their families [2, 3]. Thus, there is an urgent need to develop an effective antidementia drug, while improving cerebral pathological change with less adverse effects. Increasing evidence has shown that cerebral ischemia is a critical causative for the development of cognitive decline and dementia in the elderly, which involves multiple pathophysiological processes. Up to now, the exact pathogenic mechanisms that underlie vascular dementia are yet to be identified. The neurotransmitter dysfunction and neuron apoptosis have important roles in brain impairment and dementia progression [4, 5]. In addition, various clinical observations suggest that vascular dementia occurs as a result of poor cerebral blood circulation or oligaemia to brain areas [6]. Due to its complex etiological and pathological processes, the drug with single target is very difficult to obtain ideal results.

Traditional Chinese medicine has a lot of target spots in the brain due to its multiactive components. It could ameliorate several phenotypes of pathophysiology of vascular dementia and improve the ability of learning and memory of animals with vascular dementia [7]. Tao-Hong-Si-Wu decoction (TSD), a famous Chinese compound prescription, was first recorded in “YiZongJinJian” (a famous ancient Chinese medicine book), which consists of six Chinese herbs (Table 1). TSD has long been employed clinically to promote blood circulation to relieve women's irregular menses disorder; a previous study also showed that TSD exhibited many pharmacological activities, including antithrombotic effects through restraining the aggregation of platelet, and has obvious actions of blood-quickening and stasis-transforming [8, 9]. Furthermore, studies have shown that TSD possesses effective neuroprotective activity against MCAO-induced neuronal injury and consequent neurological deficits, probably mediated by the inhibition of HIF-1*α*, iNOS, and TNF-*α*, followed by suppression inflammatory responses, apoptosis, and platelet activation [10–12]. TSD possesses potent neuroprotective activity and it has been reported for treatment of VD [13]. However, its therapeutic mechanisms are still not well understood.

To determine the effects of TSD on vascular dementia, we employed nimodipine, an L-type voltage-dependent Ca^2+^ channel antagonist, as a positive control. Nimodipine is a common drug to treat vascular dementia [14]. Recent studies have reported that nimodipine could improve the symptoms of cognitive impairment, increase regional cerebral blood flow, reduce hippocampal inflammatory factors levels, and alleviate neuronal injury [15].

In this study, we investigated the protective effects of TSD on neuronal cells from ischemic insult and improvement on ability of learning and memory from VD, as well as its potential mechanisms.

## 2. Materials and Methods

### 2.1. Composition and Preparation of TSD

TSD consists of six medicinal plants as shown in Table 1. All the herbal drugs were purchased from Tong Ren Tang Pharm. CO. and identified by Department of Pharmacology, Anhui University of Chinese Medicine, China. The air-dried herbs were immersed in a total volume of 10 times (v/w) 75% ethanol for 6 h and then boiled for 1.5 h, and the decocted liquid was taken out. The residue was refluxed again for 1.5 h with eight times (v/w) 75% ethanol. After that, the supernatant was totally collected, filtrated, and concentrated to 1.8 g/mL. It was placed and stored in the refrigerators at 4°C until use.

### 2.2. Animals and Experimental Protocol

The experiment was performed using male Sprague-Dawley rats weighing 280–320 g, obtained from the Laboratory Animal Center of Anhui Medical University. They were housed four per cage in a standard animal room (23 ± 2°C, relative humidity at 55% ± 5%, and 12 h light-dark cycles) and given free access to food and water. The principles of laboratory animal care were followed, and the study was approved by the Ethics Committee of Anhui University of Chinese Medicine, China. The animals were acclimatized to the laboratory conditions for a week and then pretrained on the Morris water maze (MWM). Rats that failed to find the submerged platform in the circular pool within 2 minutes were excluded from further study [16]. The rats were randomly divided into six groups: sham-operated group, MCAO group, three different doses for TSD groups, and nimodipine group. The administered doses of TSD were 4.5, 9, and 18 g/kg and nimodipine was 20 mg/kg, respectively, once a day for 14 consecutive days. Rats in sham-operated and model group were administered with normal saline in the same volume. One hour after the last administration, the operation was performed.

### 2.3. Preparation of VD Model

VD model in rats were induced by the middle cerebral artery occlusion (MACO) method [17]. The animals were fasted overnight but allowed free access to water. They were then anesthetized with chloral hydrate (350 mg/kg, i.p.). Throughout the surgery, rectal temperature was maintained at 37 ± 0.5°C by a heating pad. A 4-0 silicon-coated monofilament nylon suture with a round tip was inserted through an arteriectomy in the common carotid artery just below the carotid bifurcation and then advanced into the internal carotid artery approximately 18 mm distal to the carotid bifurcation until a mild resistance was felt. After 2 h of MCAO, the filament was removed to allow reperfusion. As a control, sham-operated rats underwent identical surgery but did not have the suture inserted. After recovery from anaesthesia, the rats were returned to their cage with free access to water and food. An observer blinded to the identity of the groups assessed neurological deficits at 24 and 48 h using the forelimb akinesia test, while the spontaneous rotational test was used as a criterion for evaluating the ischemic insult [18]. Rats not showing behavioral deficits at the above time points were excluded from the study.

### 2.4. Morris Water Maze Test [19]

Rats were tested for spatial learning and memory in the Morris water maze. The test was carried out 10 days after MACO operations. The MWM consisted of a circular tank; four points around the edge of the pool were arbitrarily designated as north (N), south (S), east (E), and west (W), allowing the apparatus to be divided into 4 corresponding quadrants (NE, SE, NW, and SW). An escape platform was submerged approximately 2 cm below the water (22 ± 1°C) surface and placed in the NE quadrant of the maze. Extramaze cues consisted of laboratory furniture and lights (held constant throughout the experiment). A video camera was mounted above the center of the pool and all performance was recorded for subsequent analyses. The rats were trained for 4 consecutive days with 4 trials a day from any of the two starting points separated by 90°. The maximum trial duration was 120 s, and the intertrial interval was 60 s during which time the rat remained on the escape platform. If the rat did not find the platform within the allowed time, it was guided to the finish by observer. In each trail, the latency to escape onto the hidden platform was recorded by observer behind to experimental treatment. A 90 s probe trial was administered 24 h following the last test day distance spent in the target quadrant was recorded.

### 2.5. Histological Observation

For evaluation of histological damage, rats were killed after behavioral evaluation, by perfusion with physiological saline solution, followed by freshly prepared 4% (v/v) paraformaldehyde in 0.1 M phosphate buffered saline (PBS) buffer (pH 7.4). Brain was removed and postfixed in 4% (w/v) paraformaldehyde for 24 h. The paraffin embedded tissues were prepared, and serial sections at a thickness of 5 *μ*m were cut for HE staining. The numbers of normal neurons in the stratum pyramidal within the CA1 field were counted using an Olympus DP70 microscope (Japan) at a magnification of 400x.

### 2.6. Determination of ET-1, 5-HT, VEGF Contents, and AChE Activity in Hippocampus

Following the behavior tests, rats were anesthetized with chloral hydrate (350 mg/kg, i.p.) and sacrificed by decapitation. Hippocampus tissue was homogenized with 9 times physiological saline. After being centrifuged at 10000 rpm for 10 min, the supernatant was taken and diluted with saline. The concentrations of ET-1, 5-HT, VEGF, and activity of AChE in the supernatant obtained from the culture medium were assayed using ELISA kits according to the manufacturer's instructions (Yuanye Bio-Technology Co., Ltd, Shanghai).

### 2.7. Immunohistochemistry

Immunohistochemical staining was used to evaluate the expression of Bax and Bcl-2. Immunohistochemistry was carried out on formalin-fixed, paraffin-embedded sections. After being deparaffinized with xylene and dehydrated, the 5 *μ*m sections were quenched with 3% hydrogen peroxide (H_2_O_2_) in absolute methanol and treated with citric acid buffer (pH 6.0) in a 500 W microwave oven for 7 min for antigen retrieval. After blocking with 5% normal goat serum, the sections were incubated with primary antibody of Bax (diluted 1 : 100, Bioss) and Bcl-2 (diluted 1 : 100, Bioss) at 4°C overnight. After washing in PBS, the sections were incubated with secondary antibodies (goat-anti-rabbit, 1 : 500), and color reaction was developed using DAB (3,3′-diaminobenzidine) as the chromagen. The slides were then counterstained with hematoxylin, dehydrated using graded alcohols and xylene, and observed at 400 amplification times of light microscopy (Nikon 80i). JD801 morphology microscopic image analysis system was used for data analysis on the mean optical density.

Microvascular staining and counting: the microvasculatures in paraffin-embedded transverse sections (5 *μ*m) were visualized by staining endothelial cells with the CD34 monoclonal antibody using standard immunohistochemical staining technique. According to Weidner's microvascular counting method [20], low power light microscopy (magnification 400x) was used to scan and find three high-density microvascular regions in each section. Individual microvascular counts were conducted on a 400x field. Any brown-stained endothelial cell or endothelial cluster, clearly separate from an immediately adjacent microvascular unit, was considered a single, countable microvascular unit. The average microvessel density (MVD) of three regions was used as the final microvascular count. All quantitative analyses were performed in a blinded fashion.

### 2.8. Western Blotting

Hippocampal tissue was isolated and homogenized in lysis buffer on ice. The homogenate was centrifuged (12,000 ×g, 30 min, 4°C) and the total supernatant protein was measured by BCA assay (Beyotime). Then, total protein samples (30 *μ*g) were heated with 5x SDS sample buffer, separated by 12% SDS-PAGE, and transferred onto nitrocellulose filter (NC) membranes. After blocking (PBS containing 10% skim milk powder and 0.05% Tween 20) for 1.5 h, the blocked membranes were incubated in primary antibodies active Bax, Bcl-2, and *β*-actin (1 : 1,000) overnight at 4°C and then with the secondary antibody (HRP-conjugated goat anti-rabbit IgG) at room temperature for 1.5 h. Immunolabeling was then visualized by ECL kit (Amer cataway, NJ, USA). Digital images of protein bands were taken by Chemidoc XRS (BioRad).

### 2.9. Statistical Analysis

All of the data was performed using one way analysis of variance (ANOVA) with SPSS 11.5 and all results are expressed as mean ± S.D. Statistical significance was set at *P* < 0.05.

## 3. Results

### 3.1. TSD Ameliorates Learning and Memory Impairments Induced by MCAO

**Figure 1:**
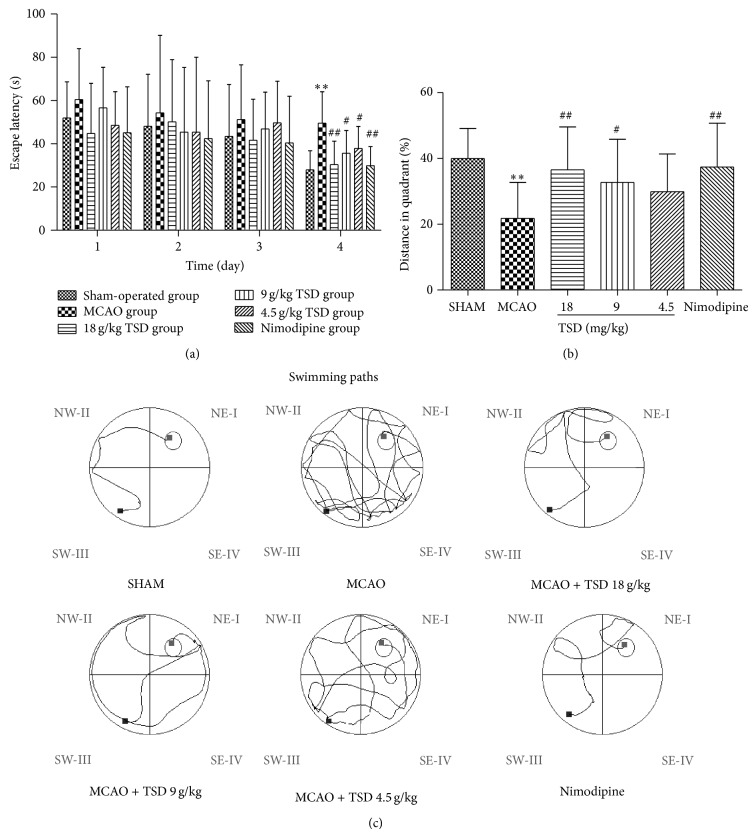
Performances of rats in Morris water maze tests. (a) The escapes latency of rats in training trials. (b) Percent distance in the target quadrant in probe trials. (c) Representative pathways in the last day of training trials. The smart cycle is the platform region. Data are presented as means ± S.D. ^**^ *P* < 0.01, ^*^ *P* < 0.05, compared with the sham operation group. ^##^ *P* < 0.01, ^#^ *P* < 0.05, compared with the model group, *n* = 12.

The effect of TSD on spatial memory in animal model of VD was shown in Figure 1.  Figure 1(a) showed that the escape latency of all groups presenting a gradually decreasing in a day-dependent manner during the former 4 days' trials. The sham-operated group rapidly learned the location of the platform and quickly reached the escape platform. In contrast, the VD model group exhibited a swimming behavior in which animals “wasted” time exploring the margin of the pool during the testing period. On the fourth day, TSD treatment groups and nimodipine group significantly shortened the escape latency by comparison with model group (*P* < 0.01 or 0.05). These results showed TSD can ameliorate the impairment of learning and memory of VD rats in dose-dependent way. In probe trials, percent time in the target quadrant of the VD model rats was shorter than the sham-operation rats, but it increased after the treatment with TSD, the TSD (18 and 9 mg/kg) and nimodipine treated rats have spent more distance over the platform compared with VD model rats (*P* < 0.01 or 0.05) (Figure 1(b)). The swimming paths of the last day were record as shown in Figure 1(c).

### 3.2. TSD Decreases Neuronal Death in the Hippocampal CA1 Region on MCAO-Induced Rats

**Figure 2:**
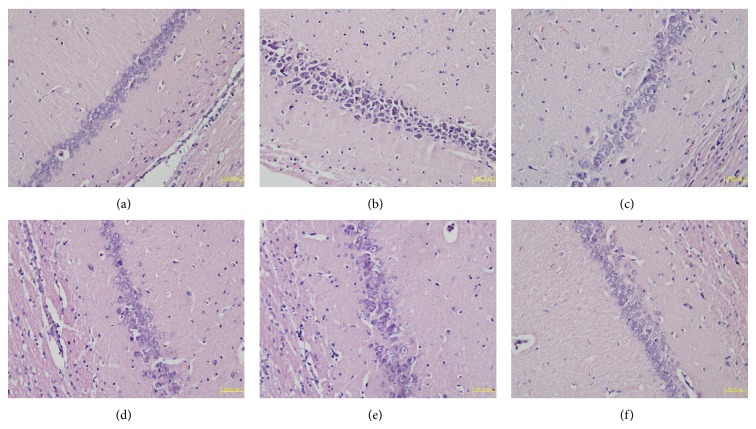
HE stains of hippocampal CA1 of brain after 14 d of MCAO. (a) Sham group; (b) model group; (c) 18 g/kg TSD; (d) 9 g/kg TSD; (e) 4.5 g/kg TSD; (f) 0.02 g/kg nimodipine. Magnification = 400.

**Figure 3:**
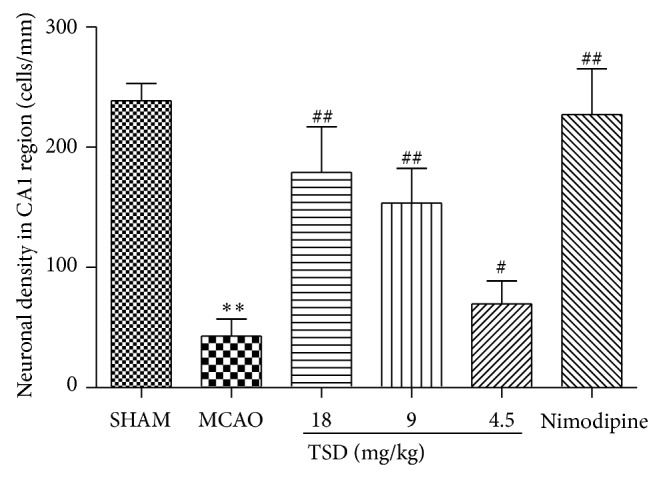
The statistical analysis of the number of surviving neurons along 1 mm liner in the middle hippocampus CA1. The results are expressed as the mean ± S.D., *n* = 6; ^*^ *P* < 0.05, ^**^ *P* < 0.01 compared with sham operation group; ^#^ *P* < 0.05, ^##^ *P* < 0.01 compared with model group.

Histological changes in the neurons of the hippocampal CA1 region were obtained by HE staining in all groups (Figure 2). In the sham group, no histopathological abnormalities were observed in the hippocampal area (Figure 2(a)). In contrast, most neurons in CA1 region in the model group appeared pyknosis with eosinophilic cytoplasm and triangulated pycnotic nucleus. The CA1 region was surrounded with necrotic neurons which exhibited pycnotic shape and condensed nuclear condensation (Figure 2(b)). After treatment with TSD and nimodipine for 14 days, the necrotic neurons markedly decreased (Figures 2(c), 2(d), 2(e), and 2(f)).  Figure 3 showed the effect of TSD on neuron density in hippocampal CA1. Surprisingly, the normal neuron density of CA1 was significantly decreased in the VD model group compared with sham-operated group, but rats treated with nimodipine and all doses of TSD dramatically enhanced neuron density (*P* < 0.01 or 0.05) (Figure 3).

### 3.3. TSD Has Effect on AChE, ET-1, 5-HT, and VEGF in the Hippocampus

Compared with the sham-operated rats, the activity of AChE and the contents of ET-1, VEGF significantly increased in model group (Table 2), whereas a significantly decrease of the content of 5-HT was found. Content of ET-1 and activity AChE in the TSD group (9 and 18 g/kg) and the nimodipine group were significantly decreased (*P* < 0.01 or 0.05 for both) compared with the model group. The enhanced content of VEGF in hippocampus was also observed in rats treated with nimodipine and TSD at doses of 18, 9 g/kg (*P* < 0.01 or 0.05 for both). Surprisingly, rats administered with TSD (4.5~18 mg/kg/day) for 14 days significantly increased the content of 5-HT (*P* < 0.01 or 0.05). TSD treatment caused dose-dependently reduced content of ET-1 and the activity of AChE.

### 3.4. TSD Increases Microvessel Density (MVD) in Hippocampus on MCAO-Induced Rats

**Figure 4:**
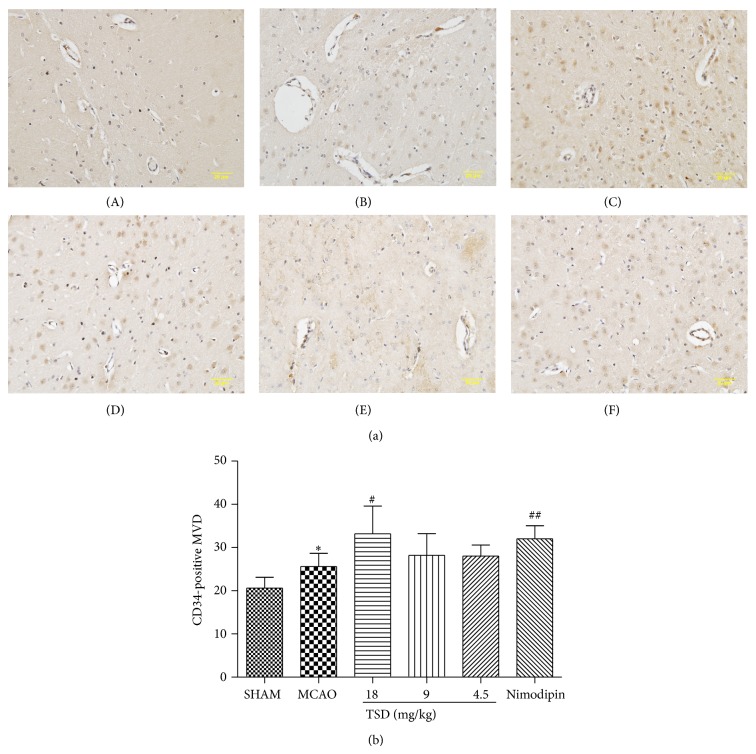
Microvessels density (MVD) in hippocampus CA1 for each group (400x magnification). (a) Immunohistochemical staining of CD34 in the ischemia area after MCAO for each group. (b) MVD was evidently increased by TSD treatment. (A) Sham group; (B) model group; (C) 18 g/kg TSD; (D) 9 g/kg TSD; (E) 4.5 g/kg TSD; (F) 0.02 g/kg nimodipine. Data were shown as mean ± S.D. (*n* = 6). ^*^ *P* < 0.05, ^**^ *P* < 0.01 compared with sham operation group; ^#^ *P* < 0.05, ^##^ *P* < 0.01 compared with model group.

MCAO-induced angiogenesis increased in the hippocampus CA1 region, and TSD treatment further increased angiogenesis after MCAO. As is shown in Figure 4, the MVD was markedly increased in the VD model group from 20.50 ± 2.26 to 26.17 ± 3.06 (*P* < 0.01) when compared with the sham-operated group. In the treatment groups with high dose of TSD, MVD markedly increased to 33.83 ± 5.95 (*P* < 0.05), in comparison to the model group (Figure 4(b)).

### 3.5. TSD Reduces Bcl-2/Bax Ratio in Hippocampus on MCAO-Induced Rats

**Figure 5:**
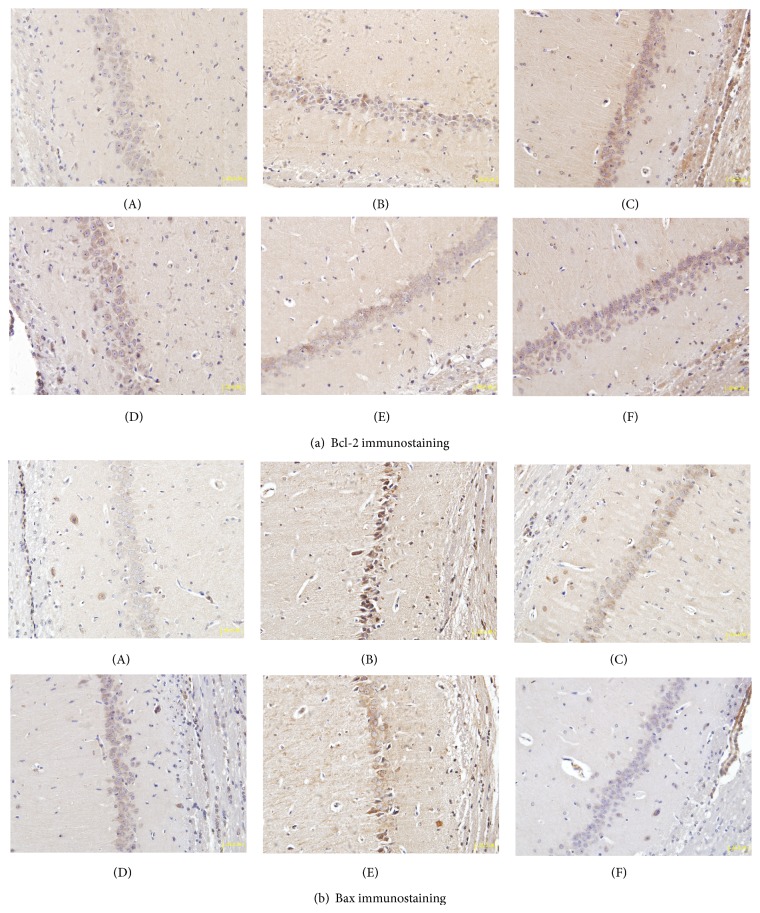
Immunohistochemistry of Bcl-2 and Bax in hippocampus CA1 of MCAO rats (magnification = 400). (a(A)) Bcl-2 immunostained tissue in sham group; (a(B)) Bcl-2 immunostained tissue in model group; (a(C)) Bcl-2 immunostained tissue in 18 g/kg TSD group; (a(D)) Bcl-2 immunostained tissue in 9 g/kg TSD group; (a(E)) Bcl-2 immunostained tissue in 4.5 g/kg TSD group; (a(F)) Bcl-2 immunostained tissue in 20 mg/kg Nimodipine group. (b(A)) Bax immunostained tissue in sham group; (b(B)) Bax immunostained tissue in model group; (b(C)) Bax immunostained tissue in 18 g/kg TSD group; (b(D)) Bax immunostained tissue in 9 g/kg TSD group; (b(E)) Bax immunostained tissue in 4.5 g/kg TSD group; (b(F)) Bax immunostained tissue in 20 mg/kg nimodipine group.

**Figure 6:**
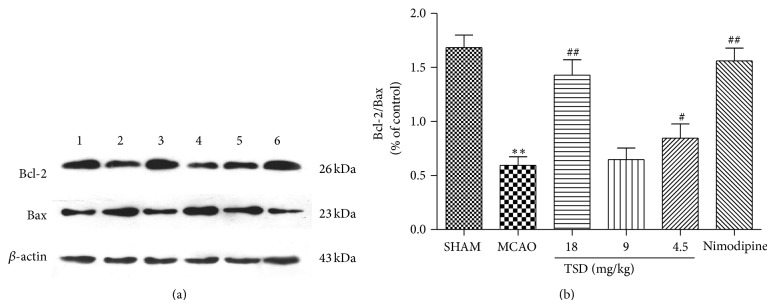
TSD reduces Bcl-2/Bax ratio. (a) Representative protein bands from western blotting for Bax and Bcl-2 in hippocampus. 1: sham group, 2: model group, 3: 18 g/kg TSD group, 4: 9 g/kg TSD group, 5: 4.5 g/kg TSD group, and 6: nimodipine group. (b) The effect of the TSD on the expression of Bax, Bcl-2, and Bcl-2/Bax of hippocampal neurons. Blots represent one of three experiments. Data were shown as mean ± S.D. (*n* = 3). ^*^ *P* < 0.05, ^**^ *P* < 0.01 compared with sham operation group; ^#^ *P* < 0.05, ^##^ *P* < 0.01 compared with model group.

To further explore molecular mechanism underlying the neuroprotective effect of TSD, we focused on two proteins implicated in apoptotic death via immunohistochemical staining. The expressions of Bax and Bcl-2 in the rat hippocampal CA1 region were delineated and quantified (Figures 5 and 6). Positive staining particles were brown in color and mainly located in the cytoplasm. Compared with the weak constitutive expression in the sham group, Bcl-2 and Bax immunostaining significantly increased (*P* < 0.01 or *P* < 0.05) in the model group. Following TSD treatment, the mean optical density of Bcl-2 was higher than those of the dementia model group (*P* < 0.05). Comparatively, a decrease in the mean optical density of Bax was seen (*P* < 0.01 or *P* < 0.05). The progressive increase of the Bcl-2/Bax ratio revealed the inhibition of TSD on neuronal apoptosis (Table 3).

To confirm these immunohistochemical data, western blotting was employed to examine the content of Bcl-2 and Bax proteins. As Figure 6 showed, the Bcl-2/Bax ratio decreased by 2.44-fold compared to the sham-operation group after MCAO treatment. However TSD treatment (18, 9, and 4.5 g/kg) altered the Bcl-2/Bax ratio to 1.43 ± 0.14, 0.65 ± 0.10, and 0.85 ± 0.13, when compared to the control group, respectively, which is consistent with the immunohistochemical data. The results of western blot also proved TSD upregulated expression of Bcl-2 and downregulated expression of Bax.

## 4. Discussion

In the present study, a VD rat model was successfully established, which has been widely accepted as an animal model of brain ischemia causing impairment in learning and memory [21]. Our works demonstrated that ischemia injury induced by MCAO resulted in a tremendous impact on the brain, and that administration of TSD significantly attenuated neurological injury by adjusting central cholinergic system, improved blood circulation of the brain, delayed neuronal cell death, and increased microvessel density in the hippocampal region. In addition, TSD suppresses MCAO-induced apoptosis via reducing Bax/Bcl-2 ratio. These beneficial effects of TSD on the ischemia were also confirmed by behavioral tasks.

It is being increasingly understood that traditional Chinese herbs can play vital roles in alleviating symptoms and healing dementia [7]. In traditional oriental medicine, many herbal drugs and prescriptions have been used clinically for the treatment of stroke, Alzheimer's disease, and vascular dementia. Chinese medicine incorporates centuries of experience in dealing with dementia. It is being increasingly understood that traditional Chinese herbs can play crucial roles in alleviating symptoms and healing vascular dementia [22]. TSD has been used for many years in traditional Chinese medical practitioners to promote blood circulation to relieve women's irregular menses disorder [23]. In addition, it can increase blood flow of the microcirculation therefore regulating diabetic neuropathies and glucocorticoid-induced avascular necrosis of the femoral head [24]. Previous research has already shown that TSD possesses potent neuroprotective activity against MCAO-induced focal cerebral ischemia* in vivo* [25]. TSD consisted of six crude extracts. These combinations contain more than 30 major active compounds such as ligustilide [26], ferulic acid [27], and safflower yellow [28], which are known to be effective as memory-improving therapeutic agents. However, herb combinations may not only act synergistically with other constituents from the same herb but may also enhance the activity of or counteract toxic effects of compounds [29]. These active compounds contained in TSD may produce better protective effects on behavioral improvement.

The MWM test employed in present study is a hippocampus dependent memory task that has been commonly employed in the evaluation of cognitive status in the ischemic brain [30]. The training trials are used to assess spatial or place learning and the probe trials to determine whether or not the animal remembers the position of the platform which reflects the memory [31]. In our experiment, we found that the escape latency was decreased with the increase in training days in both six groups which would suggest that motor function was not the underlying determinant for the prolonged latencies and learning acquisition was impaired in the VD rats. Our results suggest that VD model rats performed poorly on MWM, indicating impairment in their learning abilities and memory capabilities. Furthermore, TSD could effectively ameliorate the memory acquisition impairments. In the probe trials, TSD increased the average distance in the target quadrant. The data demonstrated that TSD improved spatial learning capabilities and reference memory. However, the mechanism of TSD's neuroprotective and antidementia effect remains largely unclear.

In behavioral test, TSD extract could improve memory impairment in rat model of vascular dementia. To further elucidate the possible mechanism associated with the recovery of memory impairment in cerebral ischemia rat, we had determined the level of cholinergic and monoamine neurotransmitter which played the vital role on learning and memory in the hippocampus. Cholinergic neurons originating in the media septum (MS) project to areas such as the cortex and hippocampus, which play a vital role in the pathophysiology of vascular dementia [32]. Lesions in these pathways result in a decrease in the ACh release and cause learning and memory dysfunction, due to the vascular dementia [33]. AChE is the most important enzymes that maintain the balance of choline level. Many studies have suggested a relationship between learning and memory functions and the AChE activity in experimental animals [4, 34]. In this research, the dosage of 18 g/kg and 9 g/kg of TSD used in the study was showed to have significant effect on enhancing the suppression of AChE activity in hippocampus; therefore, the improved memory impairment induced by TSD might occur partly via adjusting central cholinergic system ability. 5-HT is an important monoamine neurotransmitter involved in physiological, sensorimotor, and behavioral functions. Numerous studies have definitively indicated a role of 5-HT and its receptors in various aspects of cognitive functions, including learning and memory [35, 36]. Our result displayed that the content of 5-HT decreased dramatically after MCAO injury, but TSD can improve the level of 5-HT during brain ischemia. Above all TSD might prevent the impairment of learning and memory through adjusting brain neurotransmitter.

Hippocampal damage leads to cognitive impairment and has an important role in the development of vascular dementia [37]. It is well known that the hippocampus, which is a part of the brain linked to memory, is very sensitive to the brain ischemia. A large number of evidence showed that cell death of pyramidal neurons in the CA1 hippocampus causes cognitive deficits in rodents, primates, and humans [38]. Thus, pyramidal CA1 neurons of the hippocampus are essential for the learning and memory, however, CA1 neurons are especially vulnerable to brain ischemic insults, which lead to severe deficits in learning and memory after ischemic brain damage. Histopathological finding in this study showed that the CA1 area of VD rats demonstrated typical characteristics of damaged nerve cells, which has been noted as well by previous researchers [39]. In contrast, TSD plays neuroprotective role via preventing the loss of neural cells in CA1 area, suggesting that TSD has neuroprotective effects in the hippocampus.

Whether in the normal or ischemia brain, the occurrence of neurogenesis and angiogenic vessels usually accompany each other, which are important to improved functional recovery [40]. In recent years, promoted angiogenesis in cerebral regions and improved cerebral blood circulation are considered critical for treatment of VD [41]. VEGF is a key factor in promoting and harmonizing angiogenesis markedly in the ischemic brain [42] and also has neurotrophic and neuroprotective effects during the recovery stage [43–46]. Our results displayed that the VEGF content increased dramatically after ischemic brain injury; this may be a compensatory reaction of ischemia and TSD-treatment provides a significant increase in comparison with model group. ET-1 plays a central role in the regulation of blood flow, being a locally acting vasoconstrictor [47]. In the brain, ET-1 exists largely in neurons and vascular endothelial cells and has shown to be decisive for some aspects of neurological function. It has been reported that VD patients have reduced cerebral blood flow [48]. Consistently, we found that the content of ET-1 increased dramatically in the hippocampus of VD rats, but high and medium dosage of TSD significantly inhibited the amounts of ET-1. It probably means the upregulation of VEGF and downregulation of ET-1 are protective mechanisms induced to help recover blood flow and limit further brain injury.

Angiogenesis is acknowledged as a defense mechanism that can increase the oxygen and nutrition supply to the ischemia brain tissue and facilitate neurogenesis and synaptogenesis, which in turn lead to improved neurological functional recovery [49]. Microvessel density (MVD) is one of the best markers to reflect angiogenesis. In this study, we found that CD34-positive cells in the ischemic region were obviously increased in the model group when compared with the sham operated group. Additionally, MVD was increased after treatment with TSD. This result was consistent with VEGF expression outcome. These findings provided new insights into the possible regulatory mechanism of TSD for neurological recovery therapy.

Apoptosis plays a vital role in the pathophysiology of cerebral ischemia reperfusion injury and also in VD injury. Bcl-2 and Bax, two members of the Bcl-2 family, are crucial regulatory factors in apoptosis. The antiapoptotic effect of Bcl-2 occurs by prevention of cytochrome c release into the cytoplasm. By contrast, Bax belongs to proapoptotic protein, which promotes cell death by translocation into mitochondrial membrane and facilitating cytochrome c release. The Bcl-2 family maintains mitochondrial stabilization by regulating the Bcl-2/Bax balance. VD is considered a multifactorial disease, and the mechanism of neuronal apoptosis in the hippocampus is of importance [50]. Therefore, the effects of TSD on the expression of Bcl-2 and Bax in the hippocampal CA1 region were also examined. In the current study, immunohistological analysis shows that VD significantly decreased the Bcl-2 expression and increased the Bax protein expression. Compared with the sham group, the Bcl-2/Bax ratio in the model group was markedly decreased as reported in previous studies. Treatment with TSD significantly increased the Bcl-2 to Bax ratio by upregulating the expression of Bcl-2 and downregulating that of Bax in comparison to that observed in the VD model group, suggesting that TSD inhibited cerebral apoptosis after and offered ideal therapeutic approach to MCAO injury.

TSD has been widely used in China in treatment of blood stasis for centuries. In the present study, we demonstrate that TSD possesses potent neuroprotective activity against brain damage in vascular dementia. The alleviation of TSD on learning and memory deficit suggests a multifactorial mechanism probably involves adjusting neurotransmitter content, keeping cerebral vessel from injury, promoting cerebrovascular growth, and inhibiting apoptosis and ultimately stimulated the repairing mechanism of cerebral nerve injury. Our results showed that the effect of high dose of TSD is similar to nimodipine in those aspects. This study suggests that TSD may be a new promising alternative for the treatment of vascular dementia. However, further researches about possible mechanisms are still required.

## Figures and Tables

**Table 1:** Constituents of Tao-Hong-Si-Wu decoction.

Components	Part used	Amount used (g)
Shu Di Huang (*Rehmannia glutinosa* Libosch)	Root	12
Bai Shao (*Paeonialactiflora* Pallas)	Root	12
Dang Gui (*Angelicasinensis* (Oliv.) Diels)	Root	10
Chuan Xiong (*Ligusticum chuanxiong* Hort.)	Root	8
Tao Ren (*Prunus persica* (L.))	Seed	9
Hong Hua (*Carthamus tinctorius* L.)	Flower	6

**Table 2:** Effect of TSD on the activity of AChE and the contents of 5-HT, ET-1, and VEGF in hippocampus after MACO in rats.

Group	Dose (g/kg)	AChE (U/L·protein)	5-HT (ng/mL·protein)	ET-1 (ng/L·protein)	VEGF (ng/L·protein)
Sham	—	11.28 ± 0.35^**^	230.76 ± 15.89	40.65 ± 5.19^**^	108.99 ± 10.33^**^
Model	—	12.85 ± 1.09	182.31 ± 23.25	51.06 ± 3.75	125.22 ± 9.71
TSD	18	10.12 ± 0.57^**^	205.87 ± 13.53^*^	43.04 ± 6.10^**^	152.16 ± 10.71^**^
TSD	9	11.74 ± 0.98^*^	214.74 ± 16.50^**^	45.87 ± 2.26^**^	136.65 ± 10.85^*^
TSD	4.5	12.43 ± 0.54	209.36 ± 17.20^*^	49.89 ± 4.81	134.15 ± 9.94
Nimodipine	0.02	10.83 ± 0.91^**^	220.99 ± 16.45^**^	40.87 ± 5.08^**^	147.15 ± 9.92^**^

All data are mean ± S.D., *n* = 8. ^*^ *P* < 0.05, ^**^ *P* < 0.01 compared with model group.

**Table 3:** The mean optical density of Bcl-2 and Bax in hippocampus CA1 after immunostaining.

Group	Dose (g/kg)	Bcl-2 mean optical density values	Bax mean optical density values	Bcl-2/Bax
Sham	—	0.191 ± 0.007^*^	0.158 ± 0.011^**^	1.215 ± 0.110^**^
Model	—	0.202 ± 0.005	0.406 ± 0.014	0.498 ± 0.025
TSD	18	0.290 ± 0.016^**^	0.202 ± 0.016^**^	1.443 ± 0.127^**^
TSD	9	0.239 ± 0.013^**^	0.278 ± 0.012^**^	0.863 ± 0.071^**^
TSD	4.5	0.213 ± 0.011	0.374 ± 0.019^*^	0.569 ± 0.015^**^
Nimodipine	0.02	0.251 ± 0.008^**^	0.216 ± 0.017^**^	1.169 ± 0.119^**^

All data are mean ± S.D., *n* = 5. ^*^ *P* < 0.05, ^**^ *P* < 0.01 compared with model group.
